# Supplementary material for: Correlations in quantum thermodynamics: Heat, work, and entropy production
Source: Sci Rep. 2016 Oct 21;6:35568. doi: 10.1038/srep35568 (PMC5073246; doi:10.1038/srep35568)
Supplement: Supplementary Information [file srep35568-s1.pdf]

Supplementary Information for "Correlations in quantum thermodynamics: Heat, work, and entropy production"

S. Alipour[1,2], F. Benatti[3,4], F. Bakhshinezhad[2], M. Afsary[2], S. Marcantoni[3,4], and A. T. Rezakhani[2,*]

[1]School of Nano Science, Institute for Research in Fundamental Sciences (IPM), Tehran 19538, Iran
[2]Department of Physics, Sharif University of Technology, Tehran 14588, Iran
[3]Department of Physics, University of Trieste, I-34151 Trieste, Italy
[4]National Institute for Nuclear Physics (INFN), Trieste Section, I-34151 Trieste, Italy
[*]rezakhani@sharif.edu

ABSTRACT

Here we report the details of the examples.

Details of example I

State of the system

Here we obtain the exact state of the total system up to the second order in the interaction coupling λ. After calculating the interaction-picture Hamiltonian $\widetilde{H}_{\mathrm{int}}^{(\lambda)}(\tau) = U_0^{\dagger}(\tau) H_{\mathrm{int}}^{(\lambda)} U_0(\tau)$ and the corresponding evolution operator $\widetilde{U}_{\lambda}(\tau) = \mathbb{T}\mathrm{e}^{-i\int_0^{\tau}\mathrm{d}s\,\widetilde{H}_{\mathrm{int}}^{(\lambda)}(s)}$, one can read the state of the combined system from

$$\rho_{SB}^{(\lambda)}(\tau) = U_0(\tau)\widetilde{U}_{\lambda}(\tau)\rho_{SB}(0)\widetilde{U}_{\lambda}^{\dagger}(\tau)U_0^{\dagger}(\tau) \tag{1}$$

as

$$\begin{aligned}\rho_S^{(\lambda)}(\tau) =&\rho_S^{(0)}(\tau) + \lambda^2\Big\{\sigma_+\rho_S^{(0)}(\tau)\sigma_-\sum_k |f_k|^2\,|\eta(\omega_0,\omega_k,\tau)|^2\,\overline{n}(\omega_k,\beta) + \\ &+\sigma_-\rho_S^{(0)}(\tau)\sigma_+\sum_k |f_k|^2\,|\eta(\omega_0,\omega_k,\tau)|^2\big(\overline{n}(\omega_k,\beta)+1\big) \\ &-\sum_k |f_k|^2\Big(\xi^*(\omega_0,\omega_k,\omega_k,\tau)\,\rho_S^{(0)}(\tau)\sigma_+\sigma_- + \xi(\omega_0,\omega_k,\omega_k,\tau)\,\sigma_+\sigma_-\,\rho_S^{(0)}(\tau)\Big)\big(\overline{n}(\omega_k,\beta)+1\big) \\ &-\sum_k |f_k|^2\Big(\xi(\omega_0,\omega_k,\omega_k,\tau)\,\rho_S^{(0)}(\tau)\sigma_-\sigma_+ + \xi^*(\omega_0,\omega_k,\omega_k,\tau)\,\sigma_-\sigma_+\,\rho_S^{(0)}(\tau)\Big)\overline{n}(\omega_k,\beta)\Big\} + O(\lambda^3),\end{aligned} \tag{2}$$

and similarly for bath B,

$$\begin{aligned}\rho_B^{(\lambda)}(\tau) =&\rho_B^{\beta} + i\lambda\Big(\mathrm{Tr}[\rho_S(0)\sigma_+]\,\mathrm{e}^{i\omega_0\tau}\sum_k f_k^*\,\eta^*(\omega_0,\omega_k,\tau)\,[\rho_B^{\beta},\mathrm{a}_k] + \mathrm{Tr}[\rho_S(0)\sigma_-]\,\mathrm{e}^{-i\omega_0\tau}\sum_k f_k\,\eta(\omega_0,\omega_k,\tau)\,[\rho_B^{\beta},\mathrm{a}_k^{\dagger}]\Big) \\ &+\lambda^2\Big(\mathrm{Tr}[\rho_S(0)\sigma_-\sigma_+]\sum_{kk'}\Big\{f_k^* f_{k'}\,\eta^*(\omega_0,\omega_k,\tau)\eta(\omega_0,\omega_{k'},\tau)\,\mathrm{a}_k\,\rho_B^{\beta}\,\mathrm{a}_{k'}^{\dagger} \\ &-f_{k'}f_k^*\,\xi^*(\omega_0,\omega_{k'},\omega_k,\tau)\,\mathrm{e}^{i\tau(\omega_k-\omega_{k'})}\,\mathrm{a}_{k'}^{\dagger}\mathrm{a}_k\,\rho_B^{\beta} - f_{k'}^* f_k\,\xi(\omega_0,\omega_{k'},\omega_k,\tau)\,\mathrm{e}^{-i(\omega_k-\omega_{k'})\tau}\,\rho_B^{\beta}\,\mathrm{a}_k^{\dagger}\mathrm{a}_{k'}\Big\} \\ &+\mathrm{Tr}[\rho_S(0)\sigma_+\sigma_-]\sum_{kk'}\Big\{f_k f_{k'}^*\,\eta(\omega_0,\omega_k,\tau)\eta^*(\omega_0,\omega_{k'},\tau)\,\mathrm{a}_k^{\dagger}\,\rho_B^{\beta}\,\mathrm{a}_{k'} \\ &-f_{k'}^* f_k\,\xi(\omega_0,\omega_{k'},\omega_k,\tau)\,\mathrm{e}^{-i\tau(\omega_k-\omega_{k'})}\,\mathrm{a}_{k'}\mathrm{a}_k^{\dagger}\,\rho_B^{\beta} - f_{k'}f_k^*\,\xi^*(\omega_0,\omega_{k'},\omega_k,\tau)\,\mathrm{e}^{i(\omega_k-\omega_{k'})\tau}\,\rho_B^{\beta}\,\mathrm{a}_k\mathrm{a}_{k'}^{\dagger}\Big\}\Big) + O(\lambda^3),\end{aligned} \tag{3}$$

where

$$\eta(\omega_0,\omega_k,\tau) = \int_0^\tau \mathrm{d}s\, \mathrm{e}^{i(\omega_0-\omega_k)s}, \tag{4}$$

$$\xi(\omega_0,\omega_{k'},\omega_k,\tau) = \int_0^\tau \mathrm{d}s_1\, \mathrm{e}^{i(\omega_0-\omega_{k'})s_1}\,\eta^*(\omega_0,\omega_k,s_1), \tag{5}$$

$\overline{n}(\omega,\beta)$ shows the Planck distribution or the mean quanta number in a mode with frequency ω [equation (54) of the main text], and $\rho_S^{(0)}(\tau) = U_S(\tau)\rho_S(0)U_S^\dagger(\tau)$ is the unperturbed state of S, in which $U_S(\tau) = \mathrm{e}^{-i\tau H_S}$ (with $H_S = \omega_0\sigma_z/2$) is the free-system evolution.

In the continuum-ω limit, $\sum_k \to \int_0^\infty \mathrm{d}\omega$, we can find the dynamical equation of $\rho_S^{(\lambda)}(\tau)$. We differentiate the continuum version of equation (2) in which we take $\tau\to\infty$ in the integrals of the RHS (long-time limit). In the long-time, weak-coupling limit we have $\tau\to\infty$ and $\lambda\to 0$ such that $\lambda^2\tau = \text{const}$. This differentiation yields the Lindblad-type equation (51) of the main text.

Calculating thermodynamic properties

Using the following notation for the states of the system and the bath:

$$\rho_S^{(\lambda)}(\tau) = \rho_S^{(0)}(\tau) + \lambda^2\rho_S^{(2)}(\tau) + O(\lambda^3), \tag{6}$$

$$\rho_B^{(\lambda)}(\tau) = \rho_B^{\beta} + \lambda\rho_B^{(1)}(\tau) + \lambda^2\rho_B^{(2)}(\tau) + O(\lambda^3), \tag{7}$$

the effective Hamiltonians of S and B can be computed up to $O(\lambda^3)$ as

$$H_S^{(\mathrm{eff})}(\tau) = H_S + \lambda\,\mathrm{Tr}_B\left[\rho_B^{(1)}(\tau)\,H_{\mathrm{int}}^{(\lambda)}\right] - \lambda\,\alpha_S \mathrm{Tr}\left[\rho_S^{(0)}(\tau)\otimes\rho_B^{(1)}(\tau)\,H_{\mathrm{int}}^{(\lambda)}\right], \tag{8}$$

$$H_B^{(\mathrm{eff})}(\tau) = H_B + \mathrm{Tr}_S\left[\rho_S^{(0)}(\tau)\,H_{\mathrm{int}}^{(\lambda)}\right] - \lambda\,\alpha_B \mathrm{Tr}\left[\rho_S^{(0)}(\tau)\otimes\rho_B^{(1)}(\tau)\,H_{\mathrm{int}}^{(\lambda)}\right]. \tag{9}$$

We obtain

$$\mathrm{Tr}_B\left[\rho_B^{(1)}(\tau)\,H_{\mathrm{int}}^{(\lambda)}\right] = 2\lambda\sum_k |f_k|^2\left(i\rho_{10}\,\sigma_-\int_0^\tau \mathrm{d}s\,\mathrm{e}^{i\omega_k\tau}\mathrm{e}^{i(\omega_0-\omega_k)s} + \mathrm{h.c.}\right),$$

$$\mathrm{Tr}_S\left[\rho_S^{(0)}(\tau)\,H_{\mathrm{int}}^{(\lambda)}\right] = 2\lambda\sum_k\left(f_k^*\rho_{10}\mathrm{e}^{i\omega_0\tau}\mathrm{a}_k + \mathrm{h.c.}\right) =: \lambda H_B^{(1)}(\tau),$$

$$\mathrm{Tr}\left[\rho_S^{(0)}(\tau)\otimes\rho_B^{(1)}(\tau)\,H_{\mathrm{int}}^{(\lambda)}\right] = 8\lambda|\rho_{10}|^2\sum_k |f_k|^2\frac{1-\cos[(\omega_0-\omega_k)\tau]}{(\omega_0-\omega_k)} =: \lambda H_B^{(2)}(\tau),$$

where "h.c." denotes Hermitian conjugate. The energy of the bath then becomes

$$\begin{aligned}\mathbb{U}_B^{(\lambda)}(\tau) &= \mathrm{Tr}\left[\rho_B^{(\lambda)}(\tau)\,H_B^{(\mathrm{eff})}(\tau)\right]\\ &= \mathbb{U}_B^{(0)} + \lambda^2\left(-\alpha_B\mathrm{Tr}\left[\rho_B^{\beta}\,H_B^{(2)}(\tau)\right] + \mathrm{Tr}\left[\rho_B^{(1)}(\tau)\,H_B^{(1)}(\tau)\right] + \mathrm{Tr}\left[\rho_B^{(2)}(\tau)\,H_B\right]\right) + O(\lambda^3),\end{aligned} \tag{10}$$

which gives

$$\begin{aligned}\mathrm{d}\mathbb{U}_B^{(\lambda)}(\tau) =& \mathrm{d}\mathbb{Q}_B(\tau) + \mathrm{d}\mathbb{W}_B(\tau)\\ =&\lambda^2\left(\mathrm{Tr}\left[\mathrm{d}\rho_B^{(2)}(\tau)\,H_B\right] + \mathrm{Tr}\left[\mathrm{d}\rho_B^{(1)}(\tau)\,H_B^{(1)}(\tau)\right] + \mathrm{Tr}\left[\rho_B^{(1)}(\tau)\,\mathrm{d}H_B^{(1)}(\tau)\right] - \alpha_B\mathrm{Tr}\left[\rho_B^{\beta}\,\mathrm{d}H_B^{(2)}(\tau)\right]\right) + O(\lambda^3).\end{aligned} \tag{11}$$

After some straightforward algebra we can see that

$$\mathrm{Tr}\left[\mathrm{d}\rho_B^{(2)}(\tau)\,H_B\right] = 8\left[\left(\overline{n}(\omega_k,\beta)+1\right)\rho_{00} - \overline{n}(\omega_k,\beta)\rho_{11}\right]\sum_k |f_k|^2\frac{\omega_k}{(\omega_0-\omega_k)}\sin[(\omega_0-\omega_k)\tau]\,\mathrm{d}\tau, \tag{12}$$

$$\mathrm{Tr}\left[\rho_B^{(1)}(\tau)\,\mathrm{d}H_B^{(1)}(\tau)\right] = 8|\rho_{10}|^2\sum_k |f_k|^2\frac{\omega_0}{(\omega_0-\omega_k)}\sin[(\omega_0-\omega_k)\tau]\,\mathrm{d}\tau, \tag{13}$$

$$\mathrm{Tr}\left[\mathrm{d}\rho_B^{(1)}(\tau)\,H_B^{(1)}(\tau)\right] = -8|\rho_{10}|^2\sum_k |f_k|^2\frac{\omega_k}{(\omega_0-\omega_k)}\sin[(\omega_0-\omega_k)\tau]\,\mathrm{d}\tau, \tag{14}$$

$$\mathrm{Tr}\left[\rho_B^{\beta}\,\mathrm{d}H_B^{(2)}(\tau)\right] = 8|\rho_{10}|^2\sum_k |f_k|^2\sin[(\omega_0-\omega_k)\tau]\,\mathrm{d}\tau. \tag{15}$$

Hence

$$\mathrm{d}\mathbb{U}_B^{(\lambda)}(\tau) = 8\lambda^2 \sum_k |f_k|^2 \sin[(\omega_0-\omega_k)\tau]\left[|\rho_{10}|^2(1-\alpha_B) + \frac{\omega_k}{(\omega_0-\omega_k)}\left[\left(\bar{n}(\omega_k,\beta)+1\right)\rho_{00} - \bar{n}(\omega_k,\beta)\rho_{11}\right]\right]\mathrm{d}\tau + O(\lambda^3). \quad (16)$$

For the entropy we have

$$\mathrm{d}\mathbb{S}_B^{(\lambda)}(\tau) = -\mathrm{Tr}\left[\mathrm{d}\rho_B^{(\lambda)}(\tau)\,\log\rho_B^\beta\right] - \mathrm{Tr}\left[\mathrm{d}\rho_B^{(\lambda)}(\tau)\,\left(\log\rho_B^{(\lambda)}(\tau)\,-\,\log\rho_B^\beta\right)\right], \quad (17)$$

where the first term has already been computed as

$$\begin{aligned}-\mathrm{Tr}\left[\mathrm{d}\rho_B^{(\lambda)}(\tau)\,\log\rho_B^\beta\right] =& \lambda^2\beta\,\mathrm{Tr}\left[\mathrm{d}\rho_B^{(2)}(\tau)\,H_B\right] + O(\lambda^3)\\ \overset{(12)}{=}& 8\lambda^2\beta\left[\left(\bar{n}(\omega_k,\beta)+1\right)\rho_{00} - \bar{n}(\omega_k,\beta)\rho_{11}\right]\sum_k |f_k|^2\frac{\omega_k}{(\omega_0-\omega_k)}\sin[(\omega_0-\omega_k)\tau]\,\mathrm{d}\tau + O(\lambda^3). \quad (18)\end{aligned}$$

In order to evaluate the second term of equation (17) we only need to take care of the contribution of order λ. We use the following integral form for the logarithm of an operator[1]:

$$\log A = \int_0^\infty \mathrm{d}x\,\left[\frac{I}{1+x} - (xI+A)^{-1}\right], \quad (19)$$

to obtain

$$\begin{aligned}\log\rho_B^{(\lambda)}(\tau)\,-\,\log\rho_B^\beta =& \int_0^\infty \mathrm{d}x\,\left[\left(xI+\rho_B^\beta\right)^{-1} - \left(xI+\rho_B^{(\lambda)}(\tau)\right)^{-1}\right]\\ =& \lambda\int_0^\infty \mathrm{d}x\left(xI+\rho_B^\beta\right)^{-1}\rho_B^{(1)}(\tau)\left(xI+\rho_B^\beta\right)^{-1} + O(\lambda^2), \quad (20)\end{aligned}$$

where we have used the identity[1]

$$(A+B)^{-1} = A^{-1} - A^{-1}BA^{-1} + A^{-1}BA^{-1}BA^{-1} - O(B^3) \quad (21)$$

to write

$$\left(xI+\rho_B^{(\lambda)}(\tau)\right)^{-1} = \left(xI+\rho_B^\beta\right)^{-1} + \left(xI+\rho_B^\beta\right)^{-1}\left(\rho_B^\beta - \rho_B^{(\lambda)}(\tau)\right)\left(xI+\rho_B^\beta\right)^{-1} + O(\lambda^2)$$

and equation (7).

To ease notation, we introduce $O_\tau = \mathrm{a}^\dagger(h_\tau) - \mathrm{a}(h_\tau)$, with

$$\mathrm{a}(h_\tau) = i\rho_{10}\sum_k f_k^*\mathrm{e}^{i\omega_k\tau}\eta(\omega_0,\omega_k,\tau)\,\mathrm{a}_k,$$

where we have followed the shorthand introduced in equation (77) of the main text to define the vector $h_\tau = \{h_k(\tau)\}$, with $h_k(\tau) = -i\rho_{10}^* f_k\mathrm{e}^{-i\omega_k\tau}\eta^*(\omega_0,\omega_k,\tau)$. Thus we can rewrite $\rho_B^{(1)}(\tau)$ as

$$\rho_B^{(1)}(\tau) = [O_\tau\,,\,\rho_B^\beta], \quad (22)$$

whence

$$-\mathrm{Tr}\left[\mathrm{d}\rho_B^{(\lambda)}(\tau)\,\left(\log\rho_B^{(\lambda)}(\tau)\,-\,\log\rho_B^\beta\right)\right] = -\lambda^2\int_0^\infty \mathrm{d}x\,\mathrm{Tr}\left[[\mathrm{d}O_\tau\,,\,\rho_B^\beta]\left(xI+\rho_B^\beta\right)^{-1}[O_\tau\,,\,\rho_B^\beta]\left(xI+\rho_B^\beta\right)^{-1}\right] + O(\lambda^3). \quad (23)$$

Considering the spectral decomposition $\rho_B^\beta = \sum_n r_n|n\rangle\langle n|$, one can see

$$\mathrm{Tr}\left[[\mathrm{d}O_\tau\,,\,\rho_B^\beta]\left(xI+\rho_B^\beta\right)^{-1}[O_\tau\,,\,\rho_B^\beta]\left(xI+\rho_B^\beta\right)^{-1}\right] = -\sum_{n,m}\langle n|\mathrm{d}O_\tau|m\rangle\langle m|O_\tau|n\rangle\,\frac{(r_n-r_m)^2}{(x+r_n)(x+r_m)}, \quad (24)$$

which yields

$$\int_0^\infty \mathrm{d}x \, \mathrm{Tr}\left[[\mathrm{d}O_\tau\,,\rho_B^\beta]\left(xI+\rho_B^\beta\right)^{-1}[O_\tau\,,\rho_B^\beta]\left(xI+\rho_B^\beta\right)^{-1}\right] = \sum_{n,m}(r_m-r_n)\log\frac{r_n}{r_m}\langle n|\mathrm{d}O_\tau|m\rangle\langle m|O_\tau|n\rangle$$
$$= \mathrm{Tr}\left[\rho_B^\beta\left(\left[O_\tau\,,\log\rho_B^\beta\right]\mathrm{d}O_\tau+\left[\mathrm{d}O_\tau\,,\log\rho_B^\beta\right]O_\tau\right)\right]$$
$$= 2\beta\sum_k\omega_k\,\mathrm{Re}\left[h_k(\tau)\,\mathrm{d}h_k^*(\tau)\right]$$
$$= 8\beta|\rho_{10}|^2\sum_k|f_k|^2\frac{\omega_k}{(\omega_0-\omega_k)}\sin[(\omega_0-\omega_k)\tau]\,\mathrm{d}\tau.$$

Thus, noting equation (17), we obtain

$$\mathrm{d}\mathbb{S}_B^{(\lambda)}(\tau) = 8\lambda^2\beta\sum_k|f_k|^2\frac{\omega_k\sin[(\omega_0-\omega_k)\tau]}{(\omega_0-\omega_k)}\left[\left(\overline{n}(\omega_k,\beta)+1\right)\rho_{00}-\overline{n}(\omega_k,\beta)\rho_{11}-|\rho_{10}|^2\right]\mathrm{d}\tau+O(\lambda^3). \tag{25}$$

Now combining equations (16) and (25), the pseudo-temperature $T_B^{(\lambda)}(\tau)$ reads as

$$T_B^{(\lambda)}(\tau) = \frac{\mathrm{d}\mathbb{U}_B^{(\lambda)}(\tau)}{\mathrm{d}\mathbb{S}_B^{(\lambda)}(\tau)}$$
$$= \frac{1}{\beta}\frac{\sum_k|f_k|^2\frac{\omega_k\sin[(\omega_0-\omega_k)\tau]}{(\omega_0-\omega_k)}\left[\left(\overline{n}(\omega_k,\beta)+1\right)\rho_{00}-\overline{n}(\omega_k,\beta)\rho_{11}-|\rho_{10}|^2+|\rho_{10}|^2[\alpha_B(\omega_k-\omega_0)+\omega_0]/\omega_k\right]}{\sum_k|f_k|^2\frac{\omega_k\sin[(\omega_0-\omega_k)\tau]}{(\omega_0-\omega_k)}\left[\left(\overline{n}(\omega_k,\beta)+1\right)\rho_{00}-\overline{n}(\omega_k,\beta)\rho_{11}-|\rho_{10}|^2\right]}. \tag{26}$$

If we go to the continuum-ω limit, take the $\tau\to\infty$ limit, and use the identity

$$\lim_{\tau\to\infty}\frac{\sin(x\tau)}{\pi x} = \delta(x), \tag{27}$$

we obtain

$$\lim_{\tau\to\infty}T_B^{(\lambda)}(\tau) = \frac{1}{\beta}\frac{\left[\left(\overline{n}(\omega_0,\beta)+1\right)\rho_{00}-\overline{n}(\omega_0,\beta)\rho_{11}\right]}{\left[\left(\overline{n}(\omega_0,\beta)+1\right)\rho_{00}-\overline{n}(\omega_0,\beta)\rho_{11}-|\rho_{10}|^2\right]}$$
$$= \frac{1}{\beta}\left[1+\frac{|\rho_{10}|^2}{\overline{n}(\omega_0,\beta)(\rho_{00}-\rho_{11})+\rho_{00}-|\rho_{10}|^2}\right]. \tag{28}$$

Let us now study system S. Since we are interested in thermalization we consider the solution to the Lindblad equation equation (51) of the main text, which is given by

$$\rho_S^{(\lambda)}(\tau) = \frac{1}{2}\begin{bmatrix} 1+z(0)\mathrm{e}^{-\widetilde{\gamma}\tau}+\tanh(\beta\omega_0/2)\left(\mathrm{e}^{-\widetilde{\gamma}\tau}-1\right) & \left(x(0)-iy(0)\right)\mathrm{e}^{-\widetilde{\gamma}\tau/2-i\omega_0\tau} \\ \left(x(0)+iy(0)\right)\mathrm{e}^{-\widetilde{\gamma}\tau/2+i\omega_0\tau} & 1-z(0)\mathrm{e}^{-\widetilde{\gamma}\tau}-\tanh(\beta\omega_0/2)\left(\mathrm{e}^{-\widetilde{\gamma}\tau}-1\right)\end{bmatrix}, \tag{29}$$

where $\widetilde{\gamma}=\gamma\coth(\beta\omega_0/2)$ and $\left(x(0),y(0),z(0)\right)$ are the initial components of the Bloch vector. We can explicitly compute $\mathrm{d}\mathbb{S}_S^{(\lambda)}(\tau)$ using the eigenvalues of $\rho_S^{(\lambda)}(\tau)$, $(1/2)\left(1\pm\sqrt{x^2(\tau)+y^2(\tau)+z^2(\tau)}\right)$, as

$$\mathrm{d}\mathbb{S}_S^{(\lambda)}(\tau) = -\frac{1}{2}\log\left(\frac{1+\sqrt{x^2(\tau)+y^2(\tau)+z^2(\tau)}}{1-\sqrt{x^2(\tau)+y^2(\tau)+z^2(\tau)}}\right)\mathrm{d}\left(\sqrt{x^2(\tau)+y^2(\tau)+z^2(\tau)}\right)$$
$$= -\frac{1}{2}\log\left(\frac{1+\sqrt{x^2(\tau)+y^2(\tau)+z^2(\tau)}}{1-\sqrt{x^2(\tau)+y^2(\tau)+z^2(\tau)}}\right)\frac{\frac{\widetilde{\gamma}}{2}\left(x^2(\tau)+y^2(\tau)\right)-\gamma z(\tau)-\widetilde{\gamma}z^2(\tau)}{\sqrt{x^2(\tau)+y^2(\tau)+z^2(\tau)}}\mathrm{d}\tau. \tag{30}$$

The energy of this system is

$$\mathbb{U}_S^{(\lambda)}(\tau) = \mathrm{Tr}\left[\rho_S^{(\lambda)}(\tau)H_S^{(\mathrm{eff})}(\tau)\right]$$
$$= \frac{\omega_0}{2}\mathrm{Tr}\left[\rho_S^{(\lambda)}(\tau)\sigma_z\right]+\lambda\,(1-\alpha_S)\left(\mathrm{Tr}\left[\rho_S^{(\lambda)}(\tau)\sigma_+\right]\mathrm{Tr}\left[\rho_B^{(\lambda)}(\tau)\mathrm{a}(f)\right]+\mathrm{Tr}\left[\rho_S^{(\lambda)}(\tau)\sigma_-\right]\mathrm{Tr}\left[\rho_B^{(\lambda)}(\tau)\mathrm{a}^\dagger(f)\right]\right)$$
$$= \frac{\omega_0}{2}z(\tau)+2\lambda^2\,(1-\alpha_S)\,(x^2(0)+y^2(0))\mathrm{e}^{-\widetilde{\gamma}\tau}\sum_k|f_k|^2\frac{1-\cos[(\omega_0-\omega_k)\tau]}{(\omega_0-\omega_k)}+O(\lambda^3), \tag{31}$$

where we used equation (56) of the main text for $\rho_S^{(\lambda)}(\tau)$ and $\rho_B^{(\lambda)}(\tau) = \rho_B^\beta + \lambda\rho_B^{(1)}(\tau) + O(\lambda^2)$. Recalling equation (55) of the main text, the expression above can be differentiated as follows:

$$\mathrm{d}\mathbb{U}_S^{(\lambda)}(\tau) = -\frac{\omega_0}{2}\gamma \mathrm{e}^{-\widetilde{\gamma}\tau}\Big(\coth(\beta\omega_0/2)z(0)+1\Big)\mathrm{d}\tau + \frac{\gamma(1-\alpha_S)}{\pi|f(\omega_0)|^2}(x^2(0)+y^2(0))\mathrm{e}^{-\widetilde{\gamma}\tau}\sum_k |f_k|^2 \sin[(\omega_0-\omega_k)\tau]\,\mathrm{d}\tau$$
$$\overset{\omega-\text{continuum}}{=} -\frac{\omega_0}{2}\gamma \mathrm{e}^{-\widetilde{\gamma}\tau}\Big(\coth(\beta\omega_0/2)z(0)+1\Big)\mathrm{d}\tau. \tag{32}$$

As a result, the inverse pseudo-temperature becomes

$$\frac{1}{T_S^{(\lambda)}(\tau)} = -\frac{1}{2}\log\left(\frac{1+\sqrt{x^2(\tau)+y^2(\tau)+z^2(\tau)}}{1-\sqrt{x^2(\tau)+y^2(\tau)+z^2(\tau)}}\right)\frac{\frac{1}{2}\coth(\beta\omega_0/2)\big(x^2(0)+y^2(0)\big)+z(\tau)\Big(\coth(\beta\omega_0/2)z(0)+1\Big)}{\sqrt{x^2(\tau)+y^2(\tau)+z^2(\tau)}(\omega_0/2)\Big(\coth(\beta\omega_0/2)z(0)+1\Big)}, \tag{33}$$

which yields

$$\lim_{\tau\to\infty}\frac{1}{T_S^{(\lambda)}(\tau)} = \beta\left[1-\frac{\big(x^2(0)+y^2(0)\big)\coth(\beta\omega_0/2)}{2\big(z(0)+\tanh(\beta\omega_0/2)\big)}\right]. \tag{34}$$

Thus, similarly to the case of $\lim_{\tau\to 0} T_B^{(\lambda)}(\tau)$, in this case too the pseudo-temperature $T_S^{(\lambda)}(\tau)$ behaves as expected if there is no initial coherence ($\rho_{10} = 0$, or equivalently, $x(0) = y(0) = 0$).

Details of example II

If we expand $\rho_B^{(\lambda)}(\tau) =: \rho_B^\beta + \lambda\rho_B^{(1)}(\tau) + \lambda^2\rho_B^{(2)}(\tau) + O(\lambda^3)$, we obtain

$$\rho_B^{(1)}(\tau) = \langle\sigma_z\rangle_S\Big[\sum_k\big(g_k(\tau)\mathrm{a}_k^\dagger - g_k^*(\tau)\mathrm{a}_k\big), \rho_B^\beta\Big], \tag{35}$$
$$\rho_B^{(2)}(\tau) = (1/2)\Big\{\sum_{kk'}\big(g_k(\tau)\mathrm{a}_k^\dagger - g_k^*(\tau)\mathrm{a}_k\big)\big(g_{k'}(\tau)\mathrm{a}_{k'}^\dagger - g_{k'}^*(\tau)\mathrm{a}_{k'}\big), \rho_B^\beta\Big\} - \sum_k\big(g_k(\tau)\mathrm{a}_k^\dagger - g_k^*(\tau)\mathrm{a}_k\big)\rho_B^\beta\sum_{k'}\big(g_{k'}(\tau)\mathrm{a}_{k'}^\dagger - g_{k'}^*(\tau)\mathrm{a}_{k'}\big). \tag{36}$$

Since we need to compute the entropy $\mathbb{S}_B^{(\lambda)}(\tau) = -\mathrm{Tr}[\rho_B^{(\lambda)}(\tau)\log\rho_B^{(\lambda)}(\tau)]$, we shall need to calculate $\log\rho_B^{(\lambda)}(\tau)$ up to $O(\lambda^3)$. In order to do so, we use the following identity[1]:

$$\log(A_0+\lambda A_1+\lambda^2 A_2) = \log A_0 + \lambda\int_0^\infty \mathrm{d}x\,(A_0+xI)^{-1}A_1(A_0+xI)^{-1}$$
$$-\lambda^2\int_0^\infty \mathrm{d}x\Big[(A_0+xI)^{-1}A_1(A_0+xI)^{-1}A_1(A_0+xI)^{-1} - (A_0+xI)^{-1}A_2(A_0+xI)^{-1}\Big] + O(\lambda^3)$$
$$=: L_0 + \lambda L_1 + \lambda^2 L_2 + O(\lambda^3). \tag{37}$$

Replacing the terms of $\rho_B^{(\lambda)}(\tau)$ in equation (37) yields

$$L_0 = \log\rho_B^\beta, \tag{38}$$
$$L_1(\tau) = \beta\langle\sigma_z\rangle_0\sum_k\omega_k\big(g_k(\tau)\mathrm{a}_k^\dagger + g_k^*(\tau)\mathrm{a}_k\big). \tag{39}$$

Hence

$$\mathbb{S}_B^{(\lambda)}(\tau) = -\mathrm{Tr}\Big[\Big(\rho_B^\beta + \lambda\rho_B^{(1)}(\tau) + \lambda^2\rho_B^{(2)}(\tau)\Big)\Big(L_0 + \lambda L_1(\tau) + \lambda^2 L_2(\tau)\Big)\Big] + O(\lambda^3)$$
$$= -\mathrm{Tr}[\rho_B^\beta L_0] - \lambda\Big(\mathrm{Tr}[\rho_B^\beta L_1(\tau)] + \mathrm{Tr}[\rho_B^{(1)}(\tau)L_0]\Big) - \lambda^2\Big(\mathrm{Tr}[\rho_B^\beta L_2(\tau)] + \mathrm{Tr}[\rho_B^{(1)}(\tau)L_1(\tau)] + \mathrm{Tr}[\rho_B^{(2)}(\tau)L_0]\Big)$$
$$+ O(\lambda^3). \tag{40}$$

From this relation we obtain

$$\mathrm{d}\$_B^{(\lambda)}(\tau) = -\lambda\Big(\mathrm{Tr}[\rho_B^\beta\,\mathrm{d}L_1(\tau)] + \mathrm{Tr}[\mathrm{d}\rho_B^{(1)}(\tau)\,L_0]\Big) - \lambda^2\Big(\mathrm{Tr}[\rho_B^\beta\,\mathrm{d}L_2(\tau)] + \mathrm{Tr}[\mathrm{d}\rho_B^{(1)}(\tau)\,L_1(\tau)] + \mathrm{Tr}[\rho_B^{(1)}(\tau)\,\mathrm{d}L_1(\tau)] + \mathrm{Tr}[\mathrm{d}\rho_B^{(2)}(\tau)\,L_0]\Big) + O(\lambda^3). \tag{41}$$

This expression has some irrelevant (i.e., vanishing) terms. This can be seen through the identity $\mathrm{d}\$(\tau) = -\mathrm{Tr}[\mathrm{d}\rho\log\rho]$, from whence

$$\mathrm{d}\$_B^{(\lambda)}(\tau) = -\lambda\,\mathrm{Tr}[\mathrm{d}\rho_B^{(1)}(\tau)\,L_0] - \lambda^2\Big(\mathrm{Tr}[\mathrm{d}\rho_B^{(1)}(\tau)\,L_1(\tau)] + \mathrm{Tr}[\mathrm{d}\rho_B^{(2)}(\tau)\,L_0]\Big) + O(\lambda^3). \tag{42}$$

One can see from the identity $\mathrm{Tr}\big[[A,B]f(B)\big] = 0$ (for any A, B, and function f) that here

$$\mathrm{Tr}[\mathrm{d}\rho_B^{(1)}(\tau)\,L_0] \overset{(35),\,(38)}{=} 0. \tag{43}$$

Thus equation (42) reduces to

$$\begin{aligned}\mathrm{d}\$_B^{(\lambda)}(\tau) &= -\lambda^2\Big(\mathrm{Tr}[\mathrm{d}\rho_B^{(1)}(\tau)\,L_1(\tau)] + \mathrm{Tr}[\mathrm{d}\rho_B^{(2)}(\tau)\,L_0]\Big) + O(\lambda^3),\\ &= 4\beta\lambda^2(1-\langle\sigma_z\rangle_S^2)\,\mathrm{d}\Delta(\tau).\end{aligned} \tag{44}$$

References

1. F. Hiai and D. Petz, *Introduction to Matrix Analysis and Applications* (Springer, Cham, 2014).
